# Use of thoracoscopy for thoracic sympathetic nerve block in primary hyperhidrosis

**DOI:** 10.1038/s41598-023-28727-5

**Published:** 2023-01-25

**Authors:** Jung Wook Han, Seha Ahn, Jin Yong Jeong, Chan Beom Park, Eunjin Eom, Soo Seog Park

**Affiliations:** 1 Department of Thoracic and Cardiovascular Surgery, Uijeongbu St. Mary’s Hospital, College of Medicine, The Catholic University of Korea, Seoul, Republic of Korea; 2 Department of Thoracic and Cardiovascular Surgery, Eunpyeong St. Mary’s Hospital, College of Medicine, The Catholic University of Korea, Seoul, Republic of Korea; 3 Department of Thoracic and Cardiovascular Surgery, Incheon St. Mary’s Hospital, College of Medicine, The Catholic University of Korea, 56 Dongsu-Ro, Bupyeong-Gu, Incheon, 21431 Republic of Korea; 4 Department of Anesthesiology, Incheon St. Mary’s Hospital, College of Medicine, The Catholic University of Korea, Seoul, Republic of Korea

**Keywords:** Medical research, Neurology

## Abstract

Thoracic sympathetic nerve block (TSNB) has been widely used in the treatment of neuropathic pain. To reduce block failure rates, TSNB is assisted with several modalities including fluoroscopy, computed tomography, and ultrasonography. The present study describes our experience assessing the usefulness of thoracoscopy in TSNB for predicting compensatory hyperhidrosis before sympathectomy in primary hyperhidrosis. From September 2013 to October 2021, TSNB was performed under local anesthesia using a 2-mm thoracoscope in 302 patients with severe primary hyperhidrosis. Among the 302 patients, 294 were included for analysis. The target level of TSNB was T3 in almost all patients. The mean procedure time was 21 min. Following TSNB, the mean temperature of the left and right palms significantly changed from 31.5 to 35.3 °C and from 31.5 to 34.8 °C, respectively. With TSNB, primary hyperhidrosis was relieved in all patients. Pneumothorax occurred in six patients, in which no chest tube insertion was required. One patient developed hemothorax and was discharged the next day after small-bore catheter drainage. Transient ptosis developed in 10 patients and improved within a day in all patients. Our experiences showed that thoracoscopic TSNB is accurate, safe, and feasible to block the thoracic sympathetic nerve in patients with severe primary hyperhidrosis.

## Introduction

Thoracoscopic sympathectomy is the most effective and definitive treatment for severe primary hyperhidrosis when conservative treatments have failed^1–3^. However, patients are hesitant to undergo thoracoscopic sympathectomy due to the possible occurrence of postoperative compensatory hyperhidrosis, which happens in 50–90% of patients, and is particularly likely in patients with craniofacial hyperhidrosis^4,5^. Since there is no effective treatment for postoperative compensatory hyperhidrosis after definite sympathectomy, many authors have proposed several strategies including limited level sympathectomy^6^, lower level sympathectomy^7,8^, sympathetic nerve clipping^9^ and temporary thoracoscopic sympathetic block^10,11^.

Thoracic sympathetic nerve block (TSNB) can be done either in an ultrasound (US)-guided manner^12^, in a computed tomography (CT)-guided manner^13^, or thoracoscopically^10^. The US-guided and CT-guided approaches are less invasive than the thoracoscopic approach. However, these approaches appear to be less accurate than the thoracoscopic approach, which allows the surgeons to visualize a targeted sympathetic nerve in their own eyes. In 2009, we started conducting thoracoscopic TSNB under local anesthesia with a 2-mm single incision not only to reduce invasiveness but also to enhance our accurateness during the surgical procedure^11^.

The purpose of this study is to share our surgical experience with TSNB, and to examine the accuracy, safety and feasibility of this technique.

## Results

Among 302 patients with severe primary hyperhidrosis who underwent TSNB using thoracoscopy, 294 patients met the study criteria. Eight patients including seven patients with compensatory hyperhidrosis and one patient with multi-level sympathetic nerve blocks were excluded from this study. The mean age of the remaining 294 patients, including 182 males and 112 females, was 29 years (Table 1). The diagnoses were primary palmar hyperhidrosis in 258 patients (87.8%) and craniofacial hyperhidrosis in 36 patients (12.2%).

**Table 1:** Patient characteristics.

Variable	Value
Age (years)	28.8 ± 12.5
Gender	
Male	182 (62%)
Female	112 (38%)
Body mass index (kg/m^2^)	23.6 ± 3.68
Primary hyperhidrosis site	
Palmar	258 (88%)
Craniofacial	104 (12%)

Values are number (%) or mean ± standard deviation.

**Figure 1:**
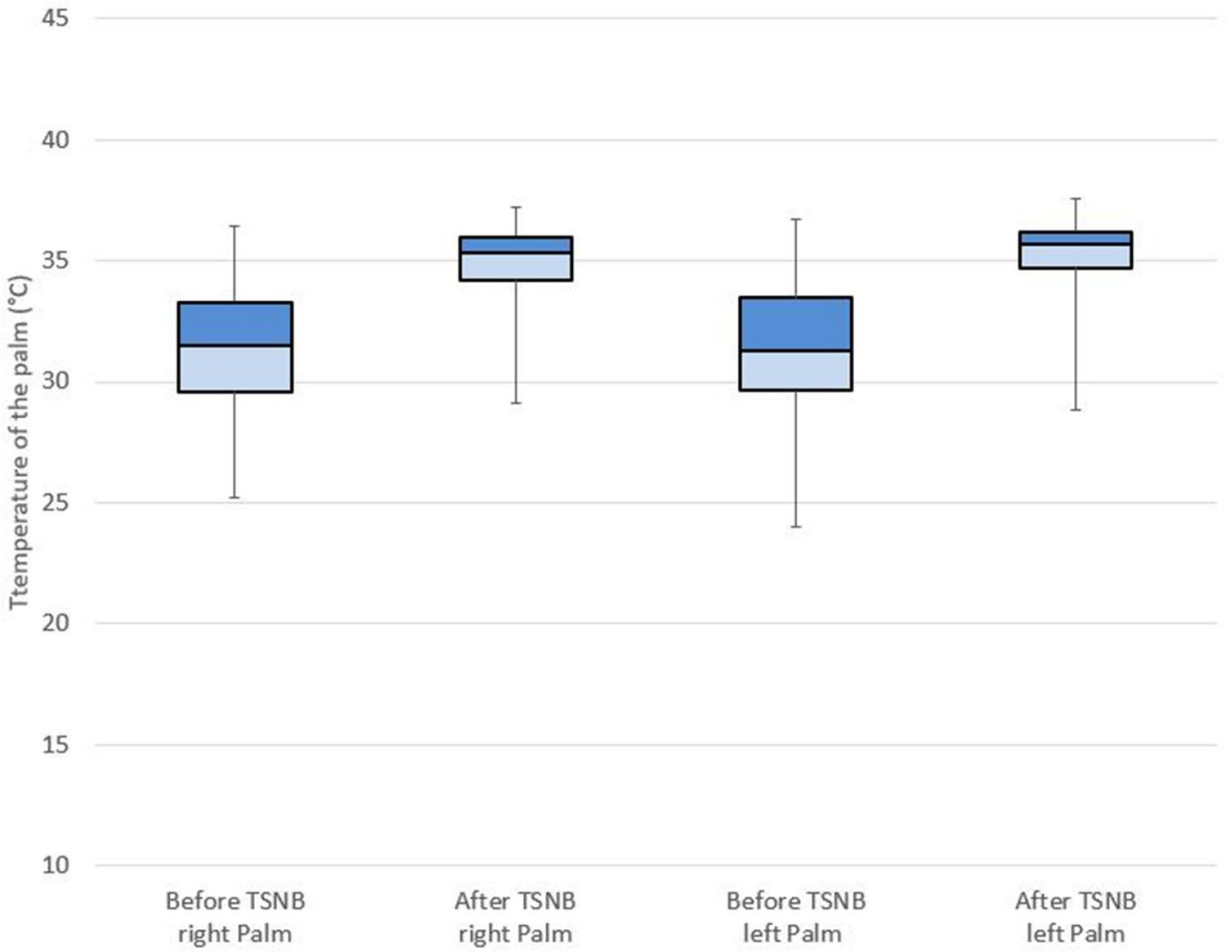
Box plot of the temperature of the palm before and after TSNB (thoracic sympathetic nerve block). The horizontal line indicates the median, the box is the interquartile range (IQR), and the whiskers extend to the upper adjacent value (large value = 75th percentile + 1.5 × IQR) and the lower adjacent value (smallest value = 25th percentile − 1.5 × IQR).

TSNBs were done at the T3 level in almost all patients. Only left or right ipsilateral TSNB was performed in 12 patients due to intraoperative pain (eight patients) or pleural adhesions (four patients). The mean procedure times of TSNB were 21 ± 8 min. The mean temperatures of the left and right palms following TSNB significantly changed from 31.5 to 35.3 °C (P < 0.0005) and from 31.5 to 34.8 °C (P < 0.0005), respectively (Table 2, Fig. 1). All patients maintained oxygen saturation levels above 90%, and there was no need for intubation during TSNB. Either palmar or craniofacial symptoms was relieved in all patients. During TSNB, there were no severe complications, such as injury of the intrathoracic organs including lung and vessels. After TSNB, pneumothorax was found in six patients (2.0%), and all the patients improved without intervention and were discharged postoperatively after one day. Hemothorax was found after TSNB with routine chest X-ray in one patient (0.3%), who was discharged the next day after the insertion of a small-bore catheter (14 G). Temporary ptosis occurred in 10 patients (3.4%) and they all improved with one day of observation.

**Table 2:** Perioperative characteristics of patients before and after TSNB.

Variable	Before TSNB	After TSNB	P value
Palm temperature (°C)			
Right	31.5 ± 2.4	34.8 ± 1.7	< 0.0005
Left	31.5 ± 2.5	35.3 ± 1.5	< 0.0005
TSNB time (minute)		21 ± 8	
Relief of symptoms		294	
Complications			
Pneumothorax		6 (2.0%)	
Hemothorax		1 (0.3%)	
Ptosis, temporary		10 (3.4%)	

Values are number (%) or mean ± standard deviation.

*TSNB* thoracic sympathetic nerve block.

## Discussion

TSNB has been widely used over the past decades as an anesthetic technique, not only to prevent persistent postoperative pain after thoracic surgery, but also to treat neuropathic pain disorders, such as complex regional pain syndrome, phantom limb pain, post-herpetic neuralgia, and ischemic vascular disease^14–17^. In recent studies, the role of TSNB has expanded to include different purposes other than pain practice. As an example, it can be applied to improve coronary microcirculation in rats with chronic heart failure. Sun and his colleagues reported that high thoracic sympathetic block improved myocardial capillary spasm and growth^18^. TSNB can also be applied as a predictive procedure for compensatory hyperhidrosis before sympathectomy in primary hyperhidrosis, based on the effect of sympathetic block on increasing skin perfusion and temperature through vasodilation at the anesthetized sites^10,11,19,20^. Miller et al.^10^ performed a temporary TSNB in 25 patients to predict whether compensatory hyperhidrosis would occur after sympathectomy in primary hyperhidrosis. All patients had temporary relief of primary hyperhidrosis after TSNB. Three patients had temporary compensatory hyperhidrosis after TSNB, and one of them experienced severe compensatory hyperhidrosis and did not proceed with sympathectomy. The remaining two patients experienced mild compensatory hyperhidrosis after TSNB and also had it after sympathectomy. Their results showed that temporary TSNB is a reversible and accurate procedure for determining compensatory hyperhidrosis after sympathectomy.

Over the past decades, there have been several approaches to blocking thoracic sympathetic nerve: the landmark-based approach, US-guided approach, CT-guided approach, fluoroscopically, and thoracoscopically^10–13,21–23^. A classic technique that does not use image guidance is a landmark-based approach that elicits a loss of resistance^21^. Other techniques include simple advancement over or under transverse process for 1 to 1.5 cm, using a nerve stimulator, pressure monitoring and X-ray direct vision^24–27^. Research examining a landmark-based approach TSNB found that the failure rate varies from 6.8 to 10%^28–30^. Like the landmark-based approach with the aid of X ray direct vision, TSNB can be conducted under imaging guidance, such as US-guidance, CT-guidance, and under fluoroscopy^12,22,23^. TSNB under fluoroscopy and the CT-guided approach results in inevitable radiation exposure and uncomfortable posture of the operator and patient during the procedures^12^. In addition, TSNB with the CT-guided approach is not practical in clinical practice. Under fluoroscopy, TSNB can be done by detecting bony surfaces as landmarks for introducing the needle for block; however, other structures such as vascular and soft tissue structures cannot be seen, which increase the risk of injuries of adjacent structures. Kim and his colleagues have reported that most patients (80%) achieved a temperature increase (≥ 1.5 °C) on the palm after TSNB under fluoroscopy at the T2 spinal level, which was superior to TSNB under the US-guided approach (20.0%)^31^. On the other hand, the US-guided approach is preferable over the previously mentioned approaches for checking the surrounding structures, including vascular and soft tissue structures, in detail to reduce complications^32,33^. A recent study by Kim and his colleagues reported that TSNB under the US-guided approach achieved a temperature increase (≥ 1.5 °C) between the ipsilateral and contralateral hands in 7 of 12 patients (58.3%), and minimized complications including pneumothorax^12^. It is true that the US-guided and CT-guided approaches are less invasive and that US-guided approach can be done at one’s bedside, while TSNB is done under thoracoscopy in the operating room.

From the perspective of accuracy and safety, thoracoscopic TSNB has the advantages of directly targeting the sympathetic nerve in our own eyes, leading to a success rate of approximately 100%^10,11^. Our results have shown that the mean temperature of the left and right palms following TSNB under thoracoscopy increased more than 1.5 °C in all patients. To further reduce the inconvenience to patients, we share here our experience of TSNB under local anesthesia with a 2-mm single incision not only to reduce invasiveness but also to enhance our accurateness during the surgical procedure. Even though we cannot directly compare our results with those of TSNB using other modalities, direct vision seems to be superior to imaging guidance. Our result has shown that pneumothorax occurred in 6 patients (n = 6/294). However, chest tube catheter drainage was not necessary, because pneumothorax was a result of not completely draining air from the chest cavity after TSNB. Only one patient developed hemothorax after TSNB, which was presumed to have occurred after removal of the trocar from the insertion site of the 2 mm surgical trocar. However, the patient was able to be discharged the next day after small-bore catheter drainage. Temporary ptosis occurred in 10 patients, which is probably caused by the block cranially extending into the sympathetic chain with the mixture injected at the T3 level. Our result has shown that TSNB under thoracoscopy is not inferior to the US-guided approach in terms of complications.

The advantages of thoracoscopic TSNB are the facts that the injection level is accurate, the injection is done under direct vision, and the procedure can be completed before the temperature change of the palm. On the other hand, thoracoscopic TSNB has several disadvantages. First, it is more invasive than the image-assisted procedure, as it requires a tiny skin incision. Second, if there is pleural adhesion, the procedure is difficult. Third, it is difficult for the patient to breathe during the procedure because it is performed under local anesthesia with the lung collapsed. Fourth, complications such as pneumothorax may occur after the procedure due to lung injury caused by thoracoscopic insertion. To predict the effect of sympathectomy for the treatment of hyperhidrosis and the occurrence of compensatory hyperhidrosis, it is recommended to show the same results after the predictive procedure. Therefore, considering the advantages and disadvantages of thoracoscopic TSNB, this modality was used considering accuracy more important than invasiveness to predict compensatory hyperhidrosis before sympathectomy in primary hyperhidrosis. However, if US-guided TSNB is not significantly different from thoracoscopic TSNB in terms of accuracy, it would be better to use US-guided manner.

The limitations of this study are the facts that it was a retrospective study, was conducted at a single institution, was not performed in patients with various diseases, and did not include a comparison with TSNB performed by other modalities. In conclusion, our experiences showed that TSNB using thoracoscopy was accurate, safe and feasible for blocking the thoracic sympathetic nerve in patients with severe primary hyperhidrosis. However, further studies are needed to compare these results with those of TSNB perfomed for other diseases and with the use of other modalities, US-guided or CT-guided manner, in the future.

## Methods

From September 2013 to October 2021, 302 consecutive patients with severe primary hyperhidrosis underwent TSNB using thoracoscopy performed by the same surgeon. The patients were included in this study who had severe primary palmar and craniofacial hyperhidrosis. We excluded patients with compensatory hyperhidrosis or recurrent hyperhidrosis who had undergone previous treatments as well as patients who required multi-level sympathetic nerve blocks due to the presence of multiple symptom sites other than palmar or craniofacial.

### Anesthesia

All patients underwent TSNB under local anesthesia in an operating room. Spontaneous respiration was maintained and supported by oxygen (3–5 L/min via mask), case by case, without endotracheal tube. Standard monitoring included electrocardiogram, non-invasive blood pressure, pulse oximetry, and respiratory rate. Temperature probes (Skin Temperature Probe D-S18A, Exacon Scientific, Roskild, Denmark) were applied on both palms.

### Surgical technique: TSNB

**Figure 2:**
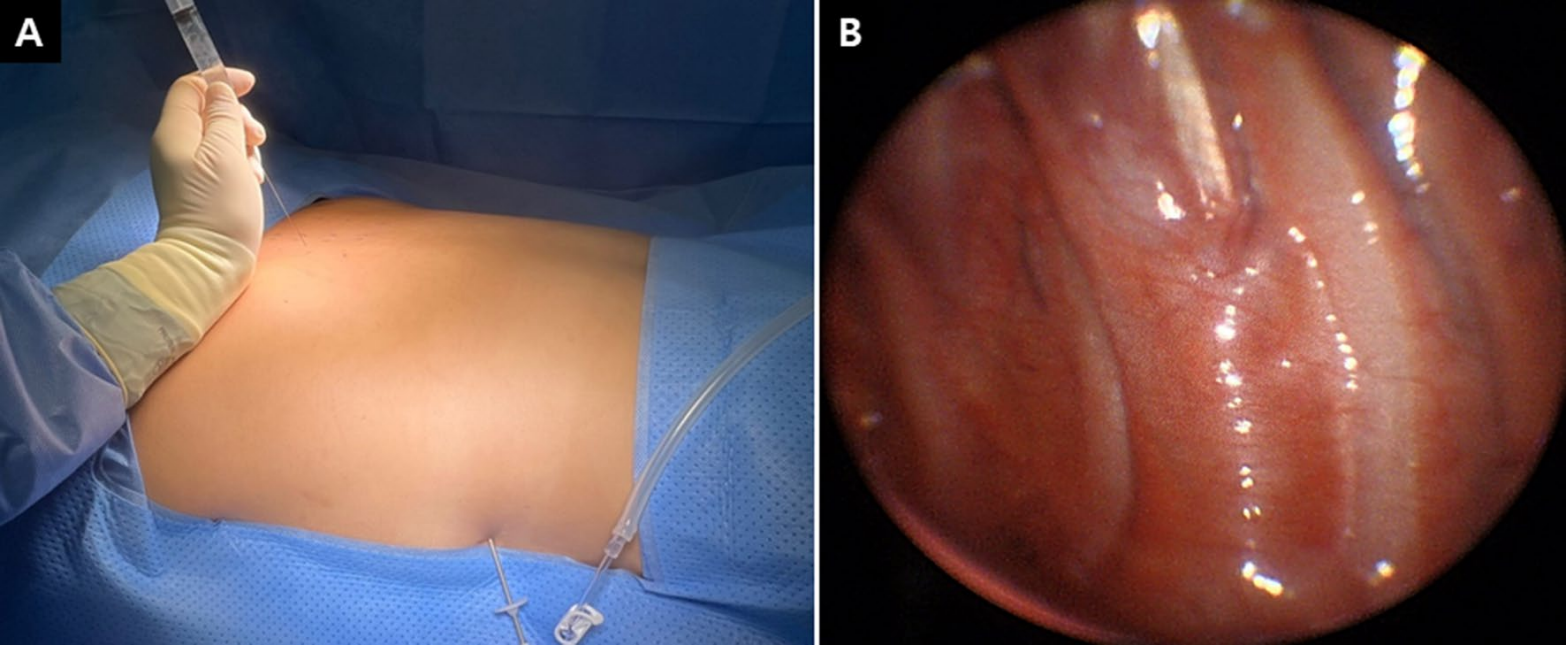
Use of thoracoscopy (**A**) for left thoracic sympathetic nerve block and (**B**) under local anesthesia while having the patient in the prone position.

The patients were placed in a prone position, and a doughnut-shaped cushion was applied to their forehead. Both arms were abducted at 90 degrees and both elbows were flexed at less than 90 degrees. A single surgical incision that was approximately 2-mm long was made at the fifth intercostal space in the mid-axillary line after local anesthesia (0.5% lidocaine) was injected from the skin to the pleura. A surgical trocar (MiniPort 2 mm, Tyco Healthcare UK Ltd., Gosport, UK) was inserted. After the insufflation of CO_2_ into the pleural space, the 2-mm thoracoscope was positioned. With visualization by thoracoscope, a spinal needle (23 Gx89mm, Hakko Co. Ltd., Chikuma, Japan) was safely introduced onto the targeted sympathetic nerve immediately above the parietal pleura through the intercostal space from the patient’s back (Fig. 2A). We used about 10 mL of a mixture (ropivacaine 37.5 mg, dexamethasone 5 mg and 0.05 mL of 0.1% epinephrine solution) that we injected through the spinal needle around both target sympathetic chains and ganglia (Fig. 2B). This local anesthetic mixture was injected slowly, followed by repeated aspiration to avoid the intravascular administration through the spinal needle around both target sympathetic chains and ganglia. After confirming that the temperature of both hands started to rise, we drained out the air through the trocar while the patient was engaging in the Valsalva maneuver, and the trocar was removed. Patients were discharged home on the day of the operation, and they did not have any complications requiring chest tube insertion after the procedure.

### Statistical analysis

We retrospectively analyzed the data of patients who underwent TSNB using thoracoscopy. Categorical variables are presented as counts and percentages. Continuous variables are presented as means with standard deviations, and they were analyzed using the dependent-sample t-test. Statistical significance was defined by P values < 0.05. All statistical analyses were performed using IBM SPSS ver. 27.0 (IBM Corp., Armonk, NY, USA),

### Ethics approval

The study was approved by the Institutional Review Board of Incheon St. Mary’s Hospital, College of Medicine, the Catholic University of Korea (IRB approval number: OC22RISI0137). The study was performed in accordance with the Declaration of Helsinki. The requirement for informed consent was waived by the Institutional Review Board of Incheon St. Mary’s Hospital, College of Medicine, the Catholic University of Korea (IRB approval number: OC22RISI0137) due to the retrospective nature of the current study.

## Data Availability

The datasets generated and analysed during the current study are available from the corresponding author on reasonable request.
